# Dr. Hamilton’s Fracture Tables

**Published:** 1852-09

**Authors:** 


*Dr. Hamilton's Fracture Tables.*—We commence in the present No. the publication of fracture tables prepared under the direction of Prof. Hamilton. These tables have been prepared with care, and embody facts which

Prof. H. has taken great pains to collect. They will form a most valuable contribution to surgical science, and especially will be found useful for reference in suits for mal-practice, a large proportion of which concern cases of fracture. We cannot doubt that the profession will hold in high estimation the service which Prof. H. has rendered by this laborious undertaking.

